# The clinical spectrum of aspergillosis in chronic obstructive pulmonary disease

**DOI:** 10.1007/s15010-022-01960-2

**Published:** 2023-01-20

**Authors:** Akaninyene Otu, Chris Kosmidis, Alexander G. Mathioudakis, Chibuike Ibe, David W. Denning

**Affiliations:** 1 Department of Microbiology, Leeds General Infirmary, Great George Street, Leeds, LS1 3EX UK; 2 Division of Evolution, Infection and Genomics, Faculty of Biology, Medicine and Health, Manchester Academic Health Science Centre, University of Manchester, Manchester, M23 9LT UK; 3 Division of Infection, Immunity and Respiratory Medicine, School of Biological Sciences, The University of Manchester, Manchester, UK; 4 North West Lung Centre, Wythenshawe Hospital, Manchester Academic Health Science Centre, Manchester University NHS Foundation Trust, Manchester, UK; 5 Department of Microbiology, Faculty of Biological Sciences, Abia State University, Uturu, Nigeria; 6 Manchester Fungal Infection Group, University of Manchester, Manchester, UK

**Keywords:** Aspergillosis, Chronic pulmonary disease, Bronchiectasis

## Abstract

Chronic obstructive pulmonary disease (COPD) is the third leading cause of death worldwide. In this review, we present the clinical spectrum and pathogenesis of syndromes caused by *Aspergillus* in COPD namely invasive aspergillosis (IA), community-acquired *Aspergillus* pneumonia, chronic pulmonary Aspergillosis and *Aspergillus* sensitisation. Some of these entities are clearly linked to COPD, while others may coexist, but are less clearly liked directly to COPD. We discuss current uncertainties as these pertain to IA in COPD cohorts and explore areas for future research in this field.

## Introduction

Chronic obstructive pulmonary disease (COPD) is the third leading cause of death worldwide [1]. It affects more than 500 million people [2] (more than 7% of the global population), posing significant morbidity, mortality, health and economic burden [3–5]. COPD is associated with chronic debilitating respiratory symptoms, including breathlessness, cough and sputum production, as well as systemic symptoms, including fatigue, muscle wasting and deconditioning [6]. COPD exacerbations, punctuating the natural history of the disease, are associated with burdensome symptoms, quality of life decline and a high mortality rate, which in the case of severe exacerbations (those requiring hospital admission) exceeds 15% within 3 months from an event [7]. These events are frequent, since each year, 22–40% of all patients with COPD experience at least one exacerbation, while 9–16% experience more than one exacerbation. Although COPD is both preventable and treatable, the clinical course is usually progressive and irreversible.

The aetiology of exacerbations is very heterogeneous, and includes bacterial [8], viral [9] or possibly fungal infections [10], enhanced eosinophilic inflammation, mechanical and environmental causes. However, in clinical practice COPD exacerbations are still managed as a single disease entity and treated with antibiotics, bronchodilators and systemic corticosteroids, that are not always required, and pose a significant treatment burden. Frequent courses of antibiotics promote antimicrobial resistance and microbial dysbiosis, while systemic corticosteroids are associated with significant side effects, that include a predisposition to infections, such as pneumonia, but also fungal infections. Better characterisation of the aetiology of COPD exacerbations in clinical practice is therefore crucial.

Considering that isolation of *Aspergillus fumigatus* from respiratory secretions is a common occurrence in COPD patients, surprisingly little is known about its significance and implications. *Aspergillus* spp. are ubiquitous fungal pathogens causing a wide range of airway disorders with *A. fumigatus* the most frequently isolated species [11]. Given their small spore size (2–3 µm) and abundance (inhaled air can contain up to 100 conidia/m^3^) [12] *Aspergillus* conidia are able to reach the lung alveoli. However, these conidia are normally promptly removed by the innate immune system of the immunocompetent host and are unable to cause disease. Immunocompromised hosts like haematological malignancy or transplant patients are at the highest risk of developing invasive aspergillosis. The airway defences of patients with COPD are less effective at killing *Aspergillus* spores [13, 14], and they can escape killing by both professional phagocytes and epithelial cells and germinate. Recently however, there has been increased interest in the role of *Aspergillus* in groups previously considered to be at low risk of aspergillosis, including COPD patients.

Various clinical syndromes related to *Aspergillus* have been described in COPD [15]. Invasive aspergillosis (IA) is well recognised in COPD patients in intensive care units (ICU) and is associated with high mortality [16, 17], partly due to delays in diagnosis. *Aspergillus* may also contribute to more indolent pathology in COPD as a subset of patients with COPD will develop chronic pulmonary aspergillosis (CPA) [18]. This entity remains underdiagnosed due to its indolent nature, but may progress to severe end-stage disease, poor quality of life and life-threatening haemoptysis. In addition, *A. fumigatus* is isolated from the sputum of patients with COPD at steady state and during exacerbations in up to a third of cases, though its contribution to the symptoms and COPD progression is unclear [15]. *Aspergillus* sensitisation or presence of *A. fumigatus* in the airways has been linked with bronchiectasis and worse forced expiratory volume in the first second (FEV1) in COPD [19]. Finally, allergic bronchopulmonary aspergillosis (ABPA) has been diagnosed in COPD patients with or without asthma, but this entity is poorly characterised, and may not be a discrete complication of COPD. [20]

In this review, we present the clinical spectrum and pathogenesis of syndromes caused by *Aspergillus* in COPD namely IA, community-acquired *Aspergillus* pneumonia, CPA and *Aspergillus* sensitisation. Some of these entities are clearly linked to COPD, while others may coexist, but are less clearly linked directly to COPD (Table 1). We discuss current uncertainties as these pertain to IA in COPD cohorts and explore areas for future research in this field.

**Table 1:** *Aspergillus* entities and linkages to COPD

Aspergillosis entity	Comments
Well documented and clearly linked	
Invasive pulmonary aspergillosis	Often a challenging diagnosis
Invasive *Aspergillus* tracheobronchitis	Usually only diagnosed in ICU or autopsy
Chronic cavitary pulmonary aspergillosis	Infrequent and often with other pulmonary disease
*Aspergillus* sensitisation	Common but of uncertain clinical significance
Community-acquired *Aspergillus* pneumonia	Probably rare, difficult to diagnose. Similar, if not identical, to sub-acute invasive pulmonary aspergillosis
*Aspergillus* nodule(s)	Leads to suspicion for carcinoma, uncertain frequency
May coexist, but less clearly linked directly to COPD	
Allergic bronchopulmonary aspergillosis	Possibly co-incident but not causally associated
*Aspergillus* bronchitis	Probably linked to coexistent bronchiectasis

## Invasive aspergillosis

IA most commonly affects people with a wide spectrum of immunosuppressive disorders [21]. In these settings, the European Organization for Research and Treatment of Cancer/Mycosis Study Group (EORTC/MSG) criteria define IA as proven, probable, or possible based on level of proof [22]. This ranges from the presence of decisive histopathological evidence of fungal invasion (proven) to a combination of clinical data and host risk factors either associated (probable) or not (possible) with the positivity of mycological criteria [at least 1 of the following: (1) cytology, direct microscopy and/or culture indicating presence of *Aspergillus* spp. in a lower respiratory tract specimen; (2) galactomannan antigen index > 0.5 in plasma/serum and/or galactomannan antigen > 0.8 in bronchoalveolar lavage (BAL) fluid. [22]

**Fig. 1:**
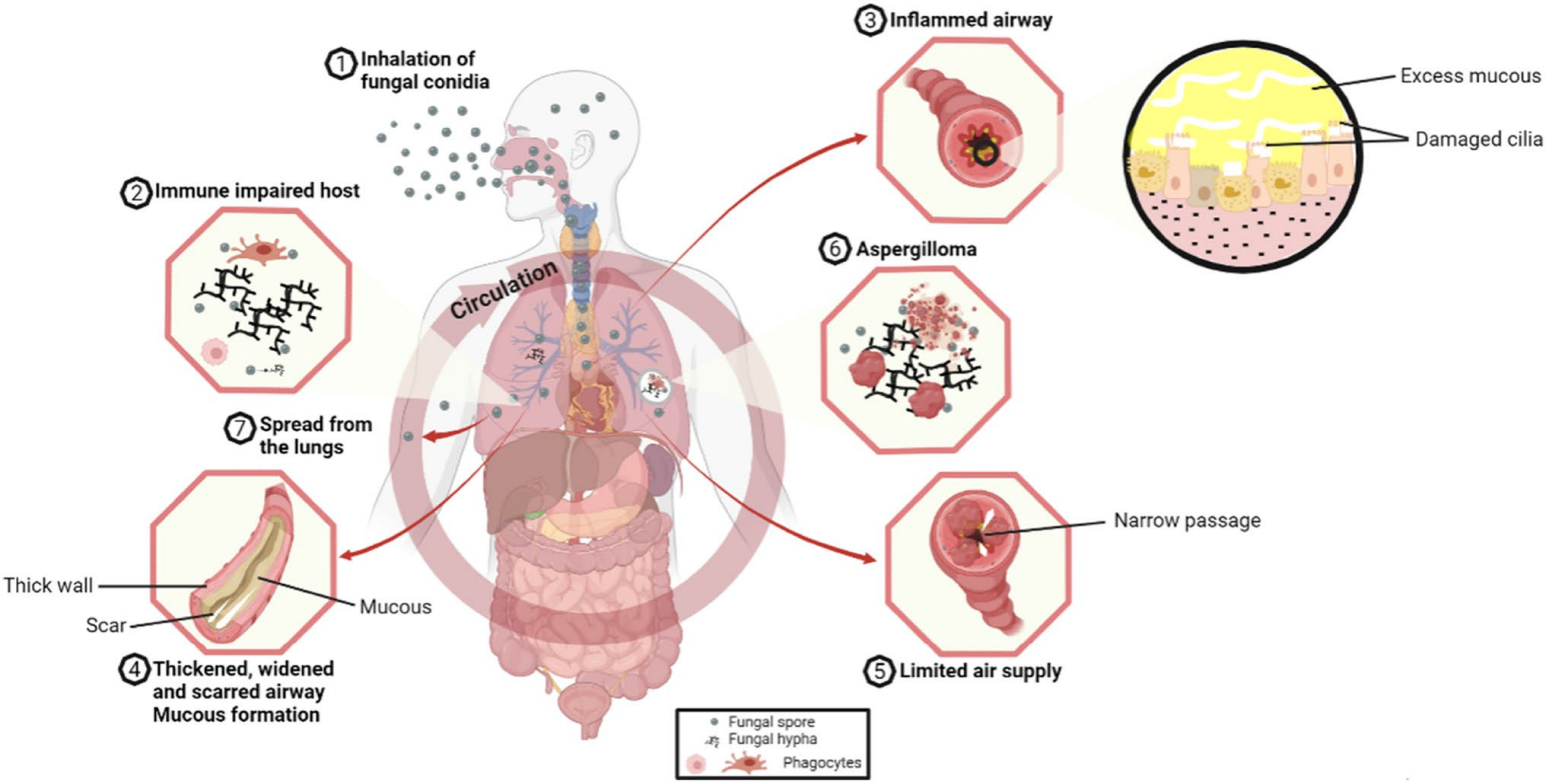
Clinical presentations of aspergillosis and associated airway obstruction

**Fig. 2:**
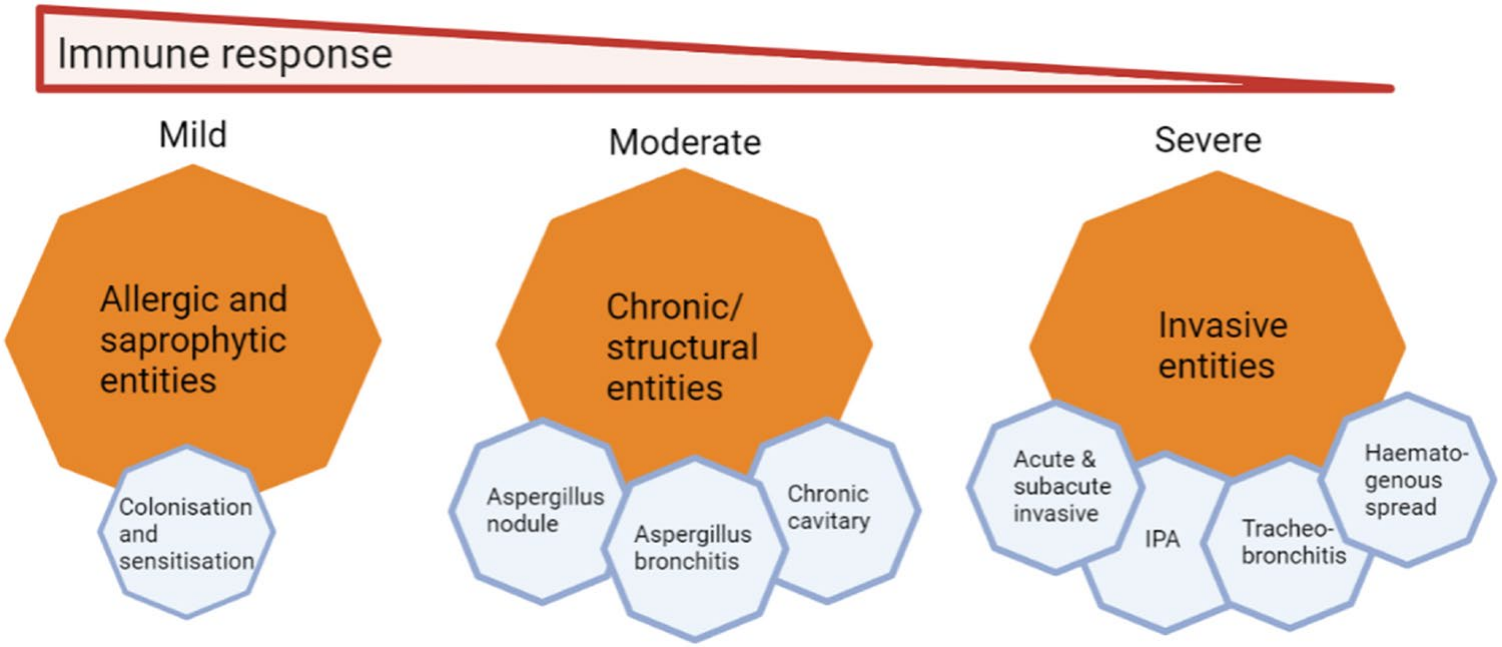
The various entities associated with *Aspergillus* pulmonary disease

Pulmonary aspergillosis is the most frequent clinical manifestation of IA, but haematogenous spread could occur resulting in the involvement of extra-pulmonary sites such as the brain and skin [23]. *Aspergillus* becomes invasive in the lungs when epithelial cells and alveolar macrophages fail to clear the conidia which germinate into branching filaments called hyphae that invade the lung tissue [24] (Fig. 1). This gives rise to several invasive entities such as tracheobronchitis, acute and sub-acute disease (Fig. 2). Acute IA occurs rapidly with clinical presentation ranging from days to 3–4 weeks. Sub-acute invasive aspergillosis (SAIA) typically occurs in patients who are not profoundly immunocompromised but may be very debilitated and usually runs a slowly progressive course over 4–12 weeks [25]. IA may be angio-invasive and non-angio-invasive with angio-invasion being especially common in neutropenic patients with invasive pulmonary aspergillosis (IPA). This angio-invasion occurs as either invasion of major proximal pulmonary arteries with resultant thrombosis and distal tissue infarction or infiltration on a vessel in the centre of a well circumscribed nodule by hyphal elements [26]. The frequency of angio-invasion in IPA in COPD is uncommon [27]. Non-angio-invasive IPA is relatively more common in corticosteroid-treated patients, including those with COPD, with micro-abscesses, nodules and acute and chronic inflammation seen more frequently than angio-invasion. In a review by Franquet et al. involving nine patients with COPD and semiinvasive aspergillosis proven at autopsy or by thoracoscopically guided lung biopsy in Spain, the radiologic findings consisted of parenchymal consolidation (*n* = 6), nodules larger than 1 cm in diameter (*n* = 3), cavitation (*n* = 2) and multiple cavitated nodules with a variable degree of central necrosis (*n* = 3) [28].

### Epidemiology of IA

IA affects primarily profoundly immunocompromised hosts, typically patients with chronic granulomatous disease, haematopoietic stem cell transplant (HSCT), prolonged neutropenia and solid organ transplant [29, 30]. However, IA in lower-risk populations is becoming increasingly recognised. These include patients with COPD [31], diabetes mellitus, renal failure, liver failure [32], high-dose corticosteroid treatment [33, 34], lung cancer [35] and those admitted to the ICU (including those with influenza and COVID-19) [31, 32, 36]. A large number of publications over the last two decades have implicated COPD as a risk factor for IA [32, 37–41].

Previous epidemiological studies may have underestimated COPD as a risk factor for IA. In a systematic review of 50 studies involving 1941 patients with IA, COPD was the underlying condition only in 26 (1.3%) [42]. This could reflect under-diagnosis of COPD. A retrospective study from Spain involving 239 patients admitted with COPD reported that IA was diagnosed in 22.1% of those with *Aspergillus* in respiratory secretions [37]. However, the prevalence of IA depends on the population studied; more critically ill patients are at greater risk of developing IA. Vandewoude et al. reported that, over a seven-year retrospective analysis, 83/172 (48%) critically ill patients with *Aspergillus* colonisation had IA [43]. In a recent systematic review, it was estimated that up to 3.9% of COPD patients admitted to hospital may have IA [2]. *Aspergillus*-colonised patients with GOLD stage 3 or 4 were more likely to have IA than those with earlier stages of COPD; among 150 patients mostly in early stages of COPD who had bronchoscopy, 17 (11%) had a positive *Aspergillus* test (culture ± galactomannan ± polymerase chain reaction [PCR]), and only 5 had probable IA [44]. Barberan et al*.* examined *Aspergillus*-colonised patients for eventual development of aspergillosis: COPD was a risk factor for developing aspergillosis and 26% of *Aspergillus*-colonised COPD patients developed aspergillosis [45].

The extremely poor prognosis of patients with COPD and IA was highlighted by Bulpa and colleagues [39]: 53 of 56 (95%) of such patients died despite optimal ICU care and antifungal treatment in 43 (77%). In an observational study involving 563 patients with IA, mortality was 38% among colonised patients, 67% in those with putative IA and 79% in those with proven IA (*p* < 0.001). In this cohort, COPD was the most common co-morbid condition (*n* = 174, 31%) [46]. Putative IPA was a strong independent risk factor for mortality in a cohort study of 50 patients admitted to ICU in France; 62.5% died in the ICU [47]. There was a statistically significant difference in the mean survival duration of patients with IA (29 days) when compared to patients without IA (86 days) in the Spanish retrospective study [37]. A retrospective cohort study of ICU patients in Belgium revealed that 127 (6.9%) out of 1850 admissions had evidence of *Aspergillus* in respiratory secretions; the majority (70%) did not have haematological malignancy; COPD was the most common diagnosis; and the observed mortality was 80% [32].

Several studies have identified risk factors for IA among patients with COPD. Rello et al*.* found corticosteroid treatment (> 20 mg of oral prednisone) and previous antibiotic use to be common among IA patients [31]. Suggested risk factors from case reports include late-stage COPD, prolonged steroid courses, viral infection and inhaled steroids [48–50]. Admission to the ICU, previous antibiotic treatment and cumulative steroid dose (> 700 mg of prednisone) in the three months prior to admission or from admission to diagnosis were independent risk factors for IA [37]. In the large series from southern China, corticosteroid exposure was only found in 13% [40], so COPD alone is important in its own right. Corticosteroid use both contributes to risk of IA and also to a worse prognosis, if not curtailed. Among 94 patients with multiple underlying diseases with IA, use of corticosteroid therapy increased the risk of dying by 10.6-fold [51].

### Clinical features and diagnosis of IA

Early clinical suspicion for IA is vital as the timely commencement of antifungals can alter the prognosis of this serious disease. In COPD patients with IA, the common clinical features include fever, dyspnoea, sputum increase, wheezing, haemoptysis and chest pain [37]. Suspecting IA from imaging in patients with COPD is challenging given the non-specific nature of the findings, but bilateral findings are seen in at least 50% of patients. In COPD patients with IA, the abnormalities on chest X-ray may include infiltrates, cavitation, nodule or consolidation. The thorax CT findings include infiltrates, nodule, consolidation, cavitation the halo sign and air crescent sign [40]. The halo sign comprises ground glass opacity due to haemorrhage that surrounds a pulmonary nodule or mass. The halo sign is less common in non-angio-invasive infection. It may be seen with or without IPA in COVID-19 Associated Pulmonary Aspergillosis (CAPA) due to the in situ infarction and endotheliopathy [52]. The air crescent sign is a late sign of IA which results from retraction of the necrotic lung from the adjacent parenchyma [53]. However, these classic clinical and radiologic features may not occur as commonly in COPD-associated IPA as in neutropenic patients [54]. 

The gold standard for the diagnosis of IA involves detection of fungi in histopathological specimens [55]. In practice, this is rarely achieved in critically ill patients, and isolation of *Aspergillus* from respiratory samples is relied upon instead. This is hampered by the relatively low positive predictive value of culture of respiratory specimens [56] and the 2–7 day turnaround time [57]. Isolation of *Aspergillus* in a patient with COPD creates diagnostic uncertainty, particularly in unwell patients and if an alternative diagnosis is not entertained. Due to these difficulties, there has been an increasing reliance on serologic and molecular diagnostic technologies. Examples of these tests include galactomannan (GM) and PCR in BAL (if the patient is intubated) and in serum, as well as beta-D-glucan (BDG) in serum. It may be that tracheal aspiration is adequate as a mycologic specimen—*Aspergillus* signals on culture and PCR are higher in sputum and bronchial samples than in BAL samples [58].

### Diagnosis with *Aspergillus* antigen detection

In immunocompromised patients, the serum GM has been established as a prognostic marker for IA. In a study involving 11 patients with grade 3 or 4 COPD, the serum GM value was found to be positive in 9 (81.8%) cases, and 5 of them (55.5%) died. Serum GM may have a role as a prognostic index in COPD cases with IPA. In a prospective study to evaluate the prognostic value of GM in the setting of severe COPD, a GM > 0.5 ng/mL, a cumulative dosage of corticosteroids > 216 mg before the ICU admission and a low creatinine clearance were predictors of poor outcome [17]. However, using a cut-off of ≥ 0.5 ng/mL for GM (Platelia, Biorad) 14 (42.4%) of the 33 patients with probable IA were positive on serum testing [37]. Further work is required to determine the utility of serum GM in diagnosing IA in COPD.

Some work has been done to compare the sensitivity of BAL fluid in the diagnosis of IPA compared to serum GM. In a prospective single-centre study involving 50 critically ill COPD patients, BAL fluid performed better than serum GM and lower respiratory tract *Aspergillus* isolation in the diagnosis of IPA. In this study, a possible cut-off value of 0.8 for GM from BAL lavage fluid was suggested in critically ill COPD patients [17].

Due to poor sensitivity of serum GM, there has been a greater reliance on GM testing of BAL fluid [59, 60]. The role of GM in serum or BAL fluid for the diagnosis of IA in patients with COPD was evaluated in a multicentre study conducted in Spain involving a total of 188 patients over a four-year period [61]. In this study, the sensitivity of BAL GM (optical density index [ODI] ≥ 1.0) was higher in immunocompromised patients compared to patients with COPD (81.8% vs 66.7%; *p*: 0.38). Among the patients with COPD, a BAL fluid ODI ≥ 0.5 provided the best performance with a sensitivity (88.9%) that equalled that of BAL fluid fungal culture. In another study involving 11 patients with IA, the optimal BAL ODI cut-off value of 1.25 was identified following analysis of receiver operating characteristics with a sensitivity of 90.9% and a specificity of 96.3% for diagnosing IA. [62]

### Diagnosis with PCR

The *Aspergillus* PCR assays holds great promise for the rapid identification of *A. fumigatus* from blood and respiratory samples such as BAL and sputum [63, 64]. Few comparisons in COPD to compare diagnostic performance of PCR and culture have been done. The data are discordant, possibly reflecting sample type and stage of patient illness. In a multicentre study that prospectively evaluated 47 mechanically ventilated patients with COPD for IA, a commercial PCR assay was positive for 10 patients (21.3%) including the two (4.2%) patients with positive culture [65]. In addition, in a study evaluating IA in 175 patients of which 91 (52.6%) had COPD, the same *Aspergillus* PCR assay on 322 lower respiratory tract samples found 15 of the 81 patients with COPD to have probable IA [66]. The sensitivity (%), specificity (%) and diagnostic odds ratio for detection of Aspergillus of this assay in these two studies (first sample/any sample) were 86.7/93, 87.6/82.4 and 48/68.75. Compared with fungal culture (median time from sample culture to visualisation of fungal growth of 3 days), PCR significantly reduced the time to diagnosis to ∼ 4 h using *Aspergillus* PCR. Therefore, PCR detection of *A. fumigatus* has the added advantage of facilitating rapid detection of azole resistance and prompt initiation of appropriate antifungal treatment.

Numerous commercial PCR assays are now available, but few have been studied explicitly in COPD patients. One of them, AsperGenius^®^, (PathoNostics, Maastricht, Netherlands), is a multiplex qPCR assay that detects azole resistance mutations in *A. fumigatus* arising from the environment [63]. The MycoGENIE^®^ (Ademtech, Pessac, France) assay is similar multiplex qPCR assay for *Aspergillus* DNA which does not detect azole resistance [63]. Another commercial PCR assay (OLM Diagnostics, Newcastle upon Tyne, UK), detects *Aspergillus terreus* as well as all *Aspergillus* species relevant as *A. terreus* is amphotericin B resistant [67].

### Diagnosis with point-of-care antigen tests

Since the early diagnosis and appropriate treatment of IA is associated with significant improvement in survival [68, 69], there has been a push to develop point-of-care (POC) tests for IA. One POC lateral flow device (LFD) (OLM Diagnostics) test utilises a mouse monoclonal antibody, JF5, which binds to a specific glycoprotein antigen produced by *Aspergillus* spp [70]. A comparison of GM, 1,3-β-D-glucan and the *Aspergillus* LFD with conventional culture was performed on 268 BAL samples from 221 patients with underlying respiratory diseases, including COPD, in Austria [71]. Probable or proven IPA was diagnosed in 14% of this study population. The LFD showed a sensitivity of 77% and specificity of 92%. The GM sensitivity and specificity was 97 and 81% for a cut-off of 0.5, and 97% and 93% for a cut-off of 1.0.

A recent comparison between a new *Aspergillus* GM lateral flow assay (LFA) (IMMY, Norman, OK, USA) and the LFD (OLM Diagnostics, Newcastle upon Tyne, UK) for the diagnosis of IA showed comparable performance as sensitivities ranged between 58 and 69%, with specificities between 68 and 75% [72]. The sensitivity increased to 81% when both tests were used in combination. It is important to note that this comparison was carried out on patients at risk for IA but without underlying haematological malignancy or neutropenia. Both the LFA and LFD tests showed promise with respect to the diagnosis of IA though targeted testing of suspected cases of IA was advocated rather than general screening of all patients [72].

Recent research on developing a POC monoclonal Ab476-based LFD for detection of urinary excreted fungal GM-like antigens is showing promise [73–75]. One advantage of this test is that it is easy to collect urine samples for this and large volumes can be tested. No data are published in patients with COPD.

There are no specific studies using POC tests for diagnosis of IA exclusively in COPD patients. It is still not clear whether the POC tests are useful for CPA or *Aspergillus* bronchitis, and they have not been trialled on sputum samples.

### Diagnosis in serum by detecting beta-D-glucan

The BDG is a key constituent of the cell wall of many pathogenic fungi and can be detected in blood of patients with invasive candidiasis, *Pneumocystis* pneumonia and IA [76, 77]. BDG detection appears to be an emerging tool for the diagnosis of IPA in COPD patients. A retrospective evaluation of laboratory results of four COPD patients in Turkey with IPA who had a BDG test (Fungitell; Associates of Cape Cod) showed that BDG was positive in 3 out 4 patients [78]. Tutar and colleagues who studied 11 COPD patients with IPA examined BDG in five patients, and this was positive in three (60%) of them [41]. However, due to a dearth of large-scale studies that evaluate BDG testing in the setting of IA, current guidelines [79, 80] do not recommend using BDG for the diagnosis of IA [81].

### Diagnosis with *Aspergillus* antibody

The clinical significance of *Aspergillus* antibody assays for the diagnosis of IA is unclear though these may have utility in the confirmation of the diagnosis of IA and for the monitoring of the treatment of IA [82]. A recent study by Yu and colleagues [83] involving 58 cases of pulmonary aspergillosis (37 IPA and 21 CPA cases) showed potential value of serum *Aspergillus* IgG antibody detection in the diagnosis of IA and CPA among a cohort of non-neutropenic patients, many with chronic pulmonary disease [83]. From the receiver operating characteristic (ROC) curves, *Aspergillus* IgG antibody detection had a higher specificity in the IPA group than in the CPA group (0.952) with *Aspergillus* IgG antibody distinguishing IPA from community-acquired bacterial pneumonia and healthy controls (sensitivity = 0.923, specificity = 0.459, cut-off value = 134.46, AUC = 0.727) and distinguishing CPA from community-acquired bacterial pneumonia and healthy controls (sensitivity = 0.952, specificity = 0.692, cut-off value = 75.46, AUC = 0.873) [83].

A cohort of 19 patients with sub-acute IA treated with voriconazole all had detectable *Aspergillus* precipitins which reduced after 6 months of therapy [84]. In a study which assessed the relationship between domestic mould exposure, *Aspergillus* biomarkers and COPD severity during acute exacerbation and at stable state, anti-*Aspergillus* antibodies (IgG and precipitins) were associated with chronic lung function alteration and/or domestic mould exposure [85]. This finding supports the consideration of indoor mould contamination and anti-*Aspergillus* antibodies kinetics in COPD management.

In a retrospective study to evaluate the utility of *Aspergillus fumigatus*-specific serum IgG and IgA (IgAG) tests for serological IPA diagnosis in 87 non-neutropenic patients in Czech Republic, GM, (1,3)-β-d-glucan and IgAG assays were found to have sensitivity/specificity/positive predictive value (PPV)/negative predictive value (NPV) of 48.8%/91.3%/83.3%/66.7%, 82.9%/73.9%/73.9%/82.9% and 75.6%/95.7%/93.9%/81.5%, respectively [86]. Of the three tests, the IgAG assay was found to have the highest specificity and PPV. Improvement was achieved by combining the GM, BG and IgAG assays.

Further work is required to explore the role of *Aspergillus* antibody assays for the diagnosis of IA in the setting of COPD.

### Treatment of IA in COPD patients

Recommendations for the treatment of IA are largely drawn from studies in immunocompromised patients, with minimal data available in COPD specifically. If voriconazole is given with prednisolone, the dose of voriconazole should be reduced by at least 30% because of a drug-drug interaction [87] and also because corticosteroids worsen outcome in IA.

## Community-acquired *Aspergillus* pneumonia and/or pneumonitis

Invasive aspergillosis in immunocompromised patients may be acquired in the community [79, 88, 89]. Recent reports of invasive aspergillosis in newly hospitalised COPD patients, many of whom, but not all, were treated with corticosteroids, emphasise the high frequency of *Aspergillus* acquisition in the community [37, 39, 40]. Virtually all cases of ABPA, *Aspergillus* bronchitis and CPA are, or presumed to be, acquired out of hospital [90]. This is hardly surprising given the ubiquitous nature of *A. fumigatus* and other species [91, 92]. It would therefore not be surprising that susceptible patients may develop community-acquired *Aspergillus* pneumonia.

**Fig. 3:**
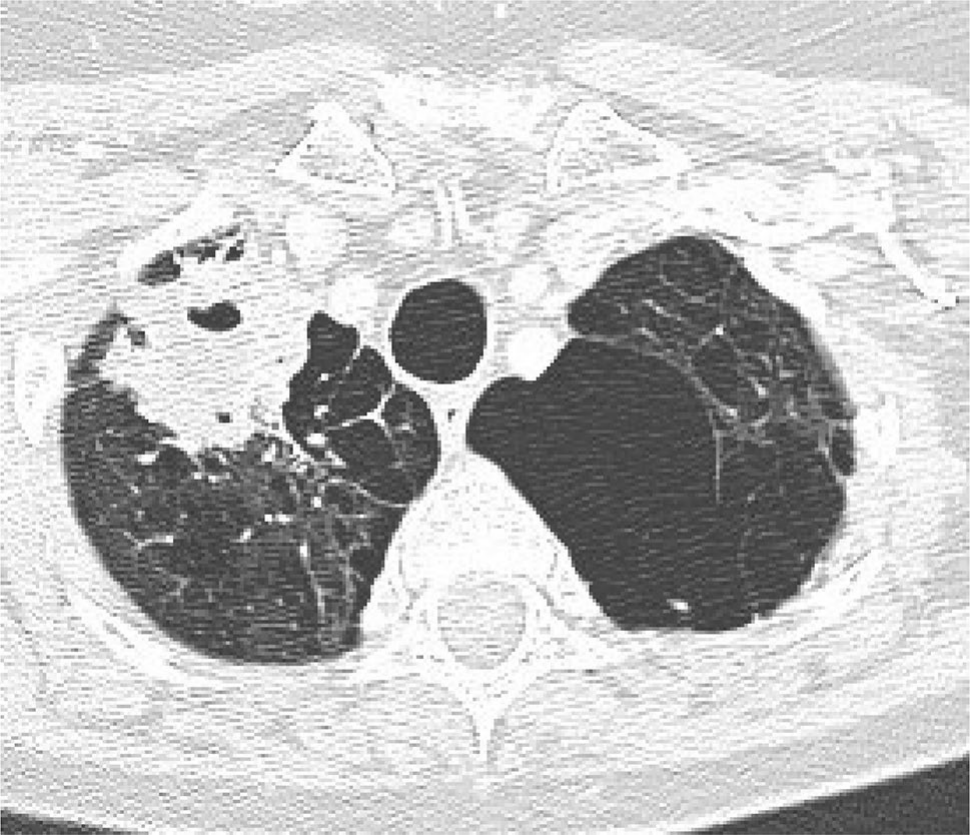
CT thorax of a female patient with asthma and significant COPD. A right upper lobe mass was found consistent with acute pneumonia. Evolution over the next few weeks and further imaging demonstrated the interval development of a fungal ball with an area of consolidation. This was consistent with a primary community-acquired *Aspergillus* pneumonia and the secondary development of chronic cavitary pulmonary aspergillosis with a fungal ball

Reports of community-acquired *Aspergillus* pneumonia and/or pneumonitis are uncommon. The terminology is also variable. There appear to be 3 common features to these reports: A remarkable exposure to airborne spores, prior influenza or other viral infection and/or underlying pulmonary disease, usually emphysema or chronic obstructive pulmonary disease (COPD). Only a few cases are described (Fig. 3).

Clancy and Nguyen presented one case and summarised the prior literature in 1998 [93]. All 12 cases they identified were infected with *A. fumigatus* and ranged in age from 14 months to 67 years. Microscopy for hyphae was positive in one patient, and sputum cultures were positive for *Aspergillus* in 7 of 10 patients (criteria for inclusion in the series). All 12 patients died, despite amphotericin B therapy in six.

Two other series require mention—both reporting a slowly progressive ‘necrotising pneumonia’ [94, 95]. Kennedy et al. report 4 cases of a necrotising pneumonia in 4 patients in Edinburgh, none immunocompromised but 2 with overt prior pulmonary disease and one a heavy smoker [94]. Binder et al. present 4 additional cases of a chronic necrotising pneumonia (chronic necrotising pulmonary aspergillosis) attributable to *A. fumigatus* or in one case *A. flavus *[95].

Since that time additional cases attributable to exposure related to damp and decomposing bark have been described [96, 97] and other cases attributable to other ‘vegetal’ [98] or decomposing plant materials [99] and gardening [100–102]. The radiological patterns described are primarily those of a miliary, bilateral diffuse pattern, with high level exposure, but unilateral, usually upper lobe cavitary disease is also well described.

Late diagnosis and lack of antifungal therapy are associated with early death and/or progressive disease. Amphotericin B therapy is probably ineffective for severe cases [93, 98, 103]. Voriconazole is recommended for treatment in the acute phase, with the addition of corticosteroids for those with overwhelming infection and/or pneumonitis, in the current state of our relative ignorance of this condition. More slowly progressive infection may respond to itraconazole.

## Chronic pulmonary aspergillosis

### Epidemiology of CPA

Chronic, allergic and saprophytic entities could also occur within the lungs following exposure to *Aspergillus* (Fig. 2). CPA is an indolent infection that affects patients with pre-existing lung disease including COPD, tuberculosis (TB), sarcoidosis, or previously treated lung cancer. CPA presents with slowly enlarging lung cavities and may remain undiagnosed resulting in poor quality of life, and secondary infections. Around 3 million people globally have CPA according to estimates, with higher prevalence in developing countries [103]. *A. fumigatus* is the commonest species causing CPA though *A. flavus, A. niger* and other rarer species have also been implicated [104]. 

A retrospective review of patients with CPA in Manchester, UK, over an 8-year period revealed that COPD was the commonest underlying condition occurring in one third of patients (42; 33.3%) with 9% having COPD as the primary underlying pulmonary disorder [18]. In a case series of 18 patients with CPA which heralded the nomenclature change for this disease, all patients had prior pulmonary disease, with COPD occurring in 10 (55.6%) [105]. In a retrospective study involving 70 CPA patients in Korea, 35 (50%) had COPD [106]. In Spain, a retrospective cohort study comprising 123 patients with COPD and respiratory isolation of *Aspergillus* spp. over a 12-year period had 7 patients with CPA in it [107]. 

COPD appears to be a strong risk factor for mortality in CPA patients. In a study that involved a cohort of 387 patients referred to the UK’s National Aspergillus Centre with CPA, survival was 86, 62 and 47% at 1, 5 and 10 years, respectively [108]. Following analysis, COPD was an indicator of mortality (hazard ratio 1.57, 1.05–2.36; *p* = 0.029). In a large study to describe the epidemiological and prognostic data of CPA patients hospitalised in France between 2008 and 2017, COPD was observed for 5 years in 46% of the 2931 CPA pts hospitalised in 2017. Emphysema was observed in 21% of patients. Among the 2605 CPA patients hospitalised in 2012, the cumulative five-year mortality rate was 41% [109]. 

### Clinical features and diagnosis of CPA

**Fig. 4:**
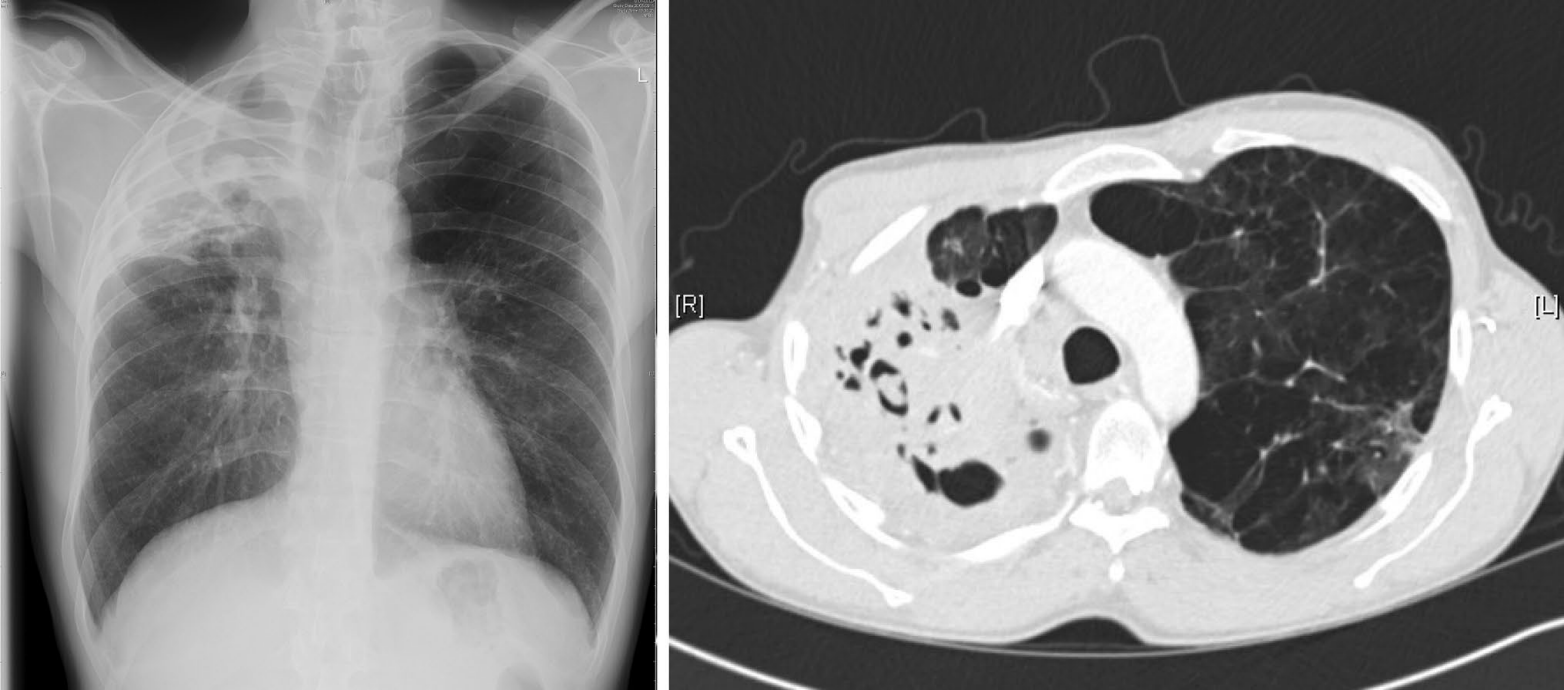
A chest radiograph and CT scan of a patient with COPD showing contraction of the right upper lobe with several large cavities and pleural thickening visible. No fungal balls are seen within the cavities. The left upper lobe is almost completely replaced by emphysematous bullae

The clinical features of CPA include chronic cough, dyspnoea, chest pain and haemoptysis which may be life-threatening. Constitutional symptoms such as sweats, anorexia, weight loss and malaise are often prominent [110]. Investigations commonly reveal thick-walled lung cavities, pleural thickening, aspergillomas and nodules (Fig. 4). Aspergillomas are made of active and dead mycelia, mucus, fibrin, epithelia cells and inflammatory cells (see Fig. 1). In COPD patients, new lung nodules are usually presumed to be malignant due to smoking history and may lead to surgery. A combination of clinical features, progressive radiological findings, and microbiological and/or serological evidence of *Aspergillus* infection is required to make the diagnosis of CPA after alternative diagnoses such as cancer or non-tuberculous mycobacterial lung disease have been excluded [111]. Guidelines on CPA endorsed by the ERS and ESCMID have been published [111].

**Fig. 5:**
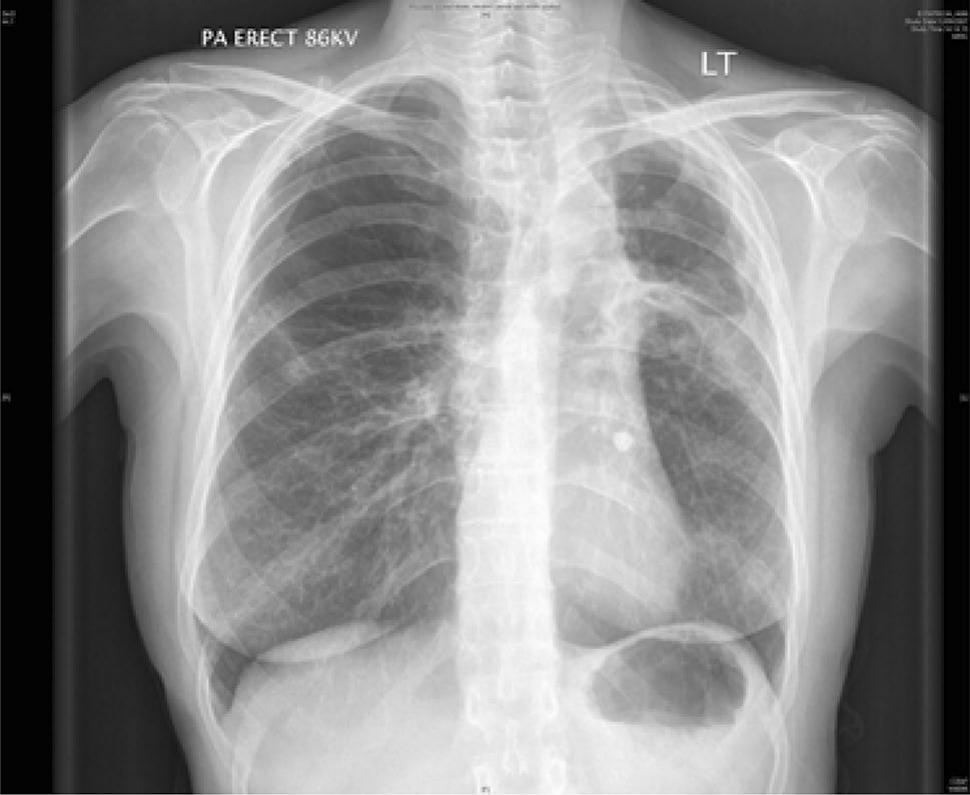
Chest X-ray of an ex-smoker (30 pack-years) patient with COPD and emphysema who developed increasing frequency of respiratory infections. Imaging shows upper lobe fibrosis with traction bronchiectasis and a positive *Aspergillus* IgG antibody test. A large cavity is visible occupying the whole left upper lobe

The progression of previous anatomic alterations within the airways such as cavitations appear to predispose to the development of CPA (Fig. 5). This may partly account for the increased risk of CPA among patients with COPD, pulmonary (TB), sarcoidosis and non-tuberculous mycobacterial infection [18]. Globally, previous pulmonary TB is the leading predisposing factor for CPA, while COPD is the most common cause in areas of low TB prevalence [112]. CPA disproportionately affects low resource settings due to high prevalence of both COPD and TB. In those settings, the diagnosis of CPA may be even more elusive due to lack of access to specialised fungal diagnostics or a CT scanner. Trend analyses have shown that the majority of deaths from COPD occur in less-developed countries [113].

### Treatment of CPA

Long-term antifungal treatment is usually required for CPA, and the triazole antifungals are the cornerstone of therapy. Treatment should be continued for at least twelve months [114] and carries response rates of 60–80%, depending on the study and antifungal agent used [106, 111, 112]. The triazoles can boost the systemic level of oral or inhaled steroids in COPD [87] thereby leading to serious complications such as Cushing’s syndrome and adrenal failure. Systemic corticosteroids should be stopped because linked to a worse clinical outcome and a threefold higher mortality [115, 116].

Surgery has a prominent role in the treatment of unilateral CPA in younger patients as it holds the promise of cure in some settings [18]. Surgical outcomes have been found to be better in patients with better lung function and localised pulmonary disease [117]. In COPD specifically, surgery may be high risk in advanced stages and is often contraindicated due to multifocal or bilateral appearance of CPA.

## *Aspergillus* nodules

*Aspergillus* nodules are an unusual form of CPA mimicking carcinoma of the lung, metastases, cryptococcal nodule, coccidioidomycosis or other rare pathogens. In immunocompromised patients, IA may present as nodules. But nodules may also occur in non-immunocompromised patients and are referred to as *Aspergillus* nodules.

**Fig. 6:**
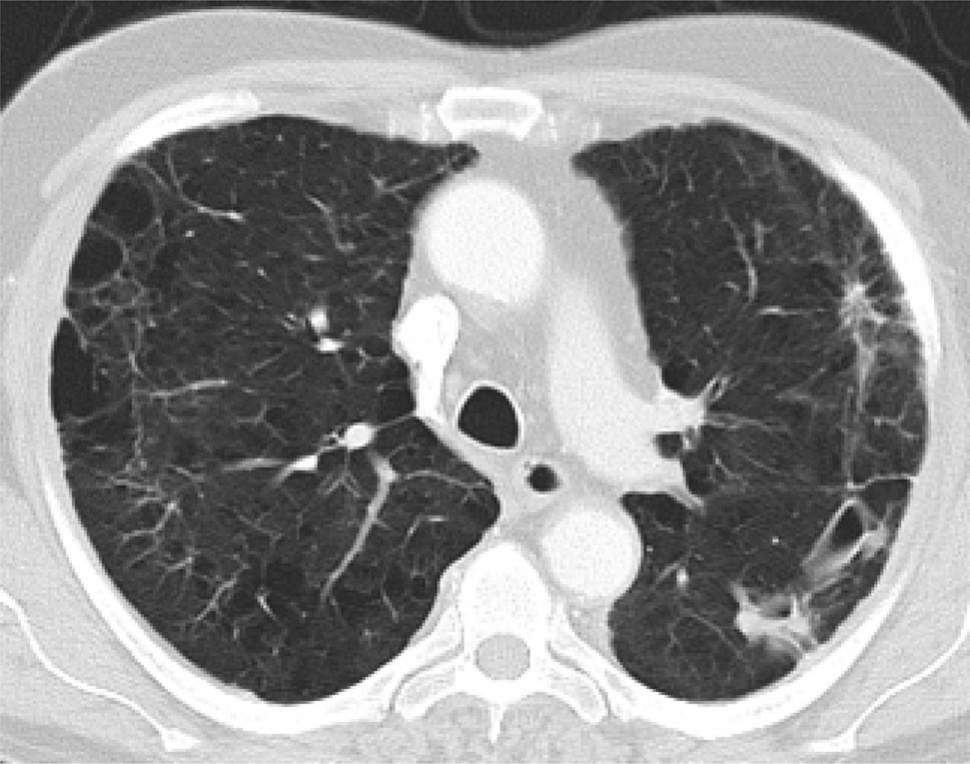
CT thorax of a long term smoker with COPD presenting with increasing shortness of breath for 20 years. CT scan shows multiple nodules on the left side, two of which had cavitated, in association with a raised *Aspergillus* IgG titre, all in keeping with chronic cavitary pulmonary aspergillosis

*Aspergillus* nodules may be single or multiple with or without cavitation, most of which are smaller than 3 cm across [118]. *Aspergillus* nodules have non-specific clinical and radiological manifestations, may be asymptomatic; the nodules are usually incidental discoveries on chest CT scan (Fig. 6). The *Aspergillus* nodules may not have distinctive features on chest CT thus making it challenging to distinguish them from malignant or other inflammatory nodules. Commoner symptoms include dyspnoea, cough, haemoptysis and weight loss [119]. This entity can only be definitively diagnosed following removal or biopsy of the nodule(s), but is suggested if some of the multiple nodules are cavitary, other infectious aetiologies are ruled out and *Aspergillus* IgG (or other microbiology) is positive.

*Aspergillus* IgG antibody testing appears to have a low sensitivity for diagnosing *Aspergillus* nodules as this was positive in only a small proportion of patients in a recent study [119]. Further research is required to clearly establish the role of *Aspergillus* IgG antibody in the diagnosis of *Aspergillus* nodules and identify other means of making the diagnosis without requiring biopsy or resection. Treatment modalities include antifungal therapy and surgical procedures such as lobectomy or sublobar resection depending on the number of nodules, their location and each patient’s lung function.

## *Aspergillus* sensitisation

*Aspergillus* sensitisation has been associated with increased severity of asthma. However, the relationship between COPD without asthma and fungal sensitisation remains unclear. COPD patients have been shown to have higher *Aspergillus* IgG (31 mg/L vs 20.7 mg/L) and more frequent *Aspergillus* sensitisation than controls (18% vs 4%) [120]. Research has shown that *Aspergillus-*sensitised individuals with COPD more often have bronchiectasis [120] as well as worse FEV1 compared to non-sensitised COPD patients (39% vs 51% predicted) [121], although this was not found in all studies [2]. The average prevalence of *Aspergillus* sensitisation in COPD from 5 studies comprising more than 1000 patients was found to be 13.6% [2]. Other studies have reported a prevalence of *Aspergillus* sensitisation in COPD of between 8.5 and 18% [122]. 

Allergic bronchopulmonary aspergillosis (ABPA) typically occurs in the setting of asthma or cystic fibrosis. In the recent past, there have been several articles have reported an association between COPD and ABPA though the exact pathophysiologic mechanism underpinning this association is yet to be elucidated. The first report of this association involved a 50‐year‐old smoker with COPD who presented with features of an exacerbation and was subsequently diagnosed with ABPA on the basis of positive *Aspergillus* skin tests (both Type I and III), elevated total serum IgE, *Aspergillus* specific IgE and IgG, eosinophilia and a positive sputum culture for *A. fumigatus* [123]. Both COPD and ABPA are characterised by the occurrence of airway inflammation, mucous hypersecretion, impaired mucociliary clearance and airflow obstruction.

A prospective case–control study involving 200 patients with COPD in India identified *Aspergillus* hypersensitivity in 17 (8.5%) of the patients with COPD as compared to none in the control group [124]. The serologic criteria for the diagnosis of ABPA were fulfilled in two (1.0%) of the patients. There have since been other case reports of ABPA occurring in patients with COPD who had neither asthma nor CF [125, 126]. This is a dynamic area of study, with uncertainty about the implications for clinical care.

## *Aspergillus* bronchitis

*Aspergillus* bronchitis is an uncommon manifestation of *Aspergillus* infection. It is a chronic superficial infection of the lower airways (trachea and bronchi) with *Aspergillus*, without important tissue invasion, lung parenchymal destruction or overt allergic response [127]. *Aspergillus* bronchitis is characterised by superficial invasion of airway mucosa by *Aspergillus* hyphae. This gives rise to mucoid impaction, production of thick tenacious sputum with bronchial plugging. *Aspergillus* IgG antibody is typically detectable in serum and *Aspergillus* spp. repeatedly detectable in sputum or bronchoscopy fluid with culture or PCR.

*Aspergillus* bronchitis is most common in those with bronchiectasis, but has also been reported to occur in patients with COPD. A retrospective analysis of 38 patients presenting with tracheobronchitis among non-neutropenic/non-transplant adult patients with at least two cultures of respiratory samples yielding *Aspergillus* spp. identified 26 (81.3%) patients with GOLD III to IV COPD [128]. A smaller series involving 17 patients with *Aspergillus* bronchitis identified 6 (35%) patients with COPD [129]. 

The various Aspergillosis entities in COPD patients and therapies are summarised in Table 2.

**Table 2:** Aspergillosis entities in COPD patients and therapies, in approximate descending order of utility and necessity (i.e. first-, second- and third-line therapies)

*Aspergillus* entities	Diagnostic tests	Treatment
Invasive pulmonary aspergillosis	Cytology, direct microscopy and/or culture of lower respiratory tract specimen, galactomannan antigen index in plasma/serum and BAL fluid, *Aspergillus* PCR on plasma/serum and BAL, chest imaging	Triazoles, amphotericin B, caspofungin or micafungin, surgical resection
Invasive *Aspergillus* tracheobronchitis	Sputum and BAL fluid culture or PCR, *Aspergillus* IgG in serum, serum IgE, chest imaging	Triazoles
Chronic cavitary pulmonary aspergillosis	*Aspergillus* IgG or precipitins in plasma/serum, *Aspergillus* antigen or PCR in respiratory fluids, microscopy/histology, chest imaging	Triazoles, surgery, amphotericin B. micafungin
Community-acquired *Aspergillus* pneumonia	*Aspergillus* IgG or precipitins in plasma/serum	Triazoles
*Aspergillus* nodule(s)	*Aspergillus* IgG or precipitins in plasma/serum, *Aspergillus* antigen or PCR in respiratory fluids, histology, chest imaging	Excision biopsy, triazoles
*Aspergillus* sensitisation	Serum IgE to *A.* *fumigatus* serum precipitins, total IgE, skin prick test or RAST test	None, triazoles, nebulised amphotericin B

## Bronchiectasis

Bronchiectasis is a chronic airways syndrome usually defined in the presence of permanent airway dilatation (often demonstrated on high-resolution CT scanning), variable mucociliary clearance, recurrent airway symptoms of daily sputum production and episodic infective exacerbations [130–132]. 

The common manifestations of *Aspergillus* disease in bronchiectasis include CPA, APBA pulmonary aspergilloma and IA [133]. *Aspergillus* diseases such as ABPA may be complicated by the development of bronchiectasis whereas patients with already established bronchiectasis and lung architectural disruption can develop *Aspergillus*-related diseases such as aspergillomas on top of post-TB bronchiectasis [134]. Bronchiectasis and COPD are reported to coexist in 20–60% of cases, and coexisting disease is linked to greater symptomatology, reduced therapeutic options and poorer prognosis when compared to bronchiectasis or COPD alone [135–138]. Misdiagnosis of the two conditions frequently occur with computed tomography (CT) without high-resolution algorithms being identified as the main cause of under-diagnosis of bronchiectasis [134]. 

Bronchiectasis does share some pathogenic similarities with COPD. In both diseases, chronic bronchial infection by pathogenic microorganisms and consequent chronic inflammation with remodelling of the airways leads to impairment of local defence mechanisms. This ultimately results in the persistence of microorganisms such as *Aspergillus* in the bronchial tree despite treatment [139]. In bronchiectasis, *Aspergillus* infection of the airways is thought to directly damage the airways and impair mucociliary clearance. In normal (or asthmatic/COPD) airways in susceptible individuals, it has been theorised the *Aspergillus* proteases upregulate mucous production and/or drive a Th2 phenotype thus drive airway inflammation towards frank bronchiectasis [130]. 

## Role of *Aspergillus* colonisation in COPD

The isolation of *Aspergillus* from respiratory secretions of COPD patients is common and usually dismissed as representing mere colonisation or contamination, especially outside the setting of critical illness and in the absence of imaging findings suggestive of IA or CPA. However, several studies attempted to assess its significance [27]. A prospective study conducted across four hospitals in Spain involving 240 hospitalised patients with a severe COPD exacerbation reported a 16.6% prevalence of *Aspergillus* spp. isolation on admission and 14.1% at 1-year follow-up. No patients were found to have IA. Those who isolated *Aspergillus* were more likely to have had an exacerbation in the preceding year and to also have other pathogens and especially *Pseudomonas aeruginosa* in sputum [140]. *Aspergillus* isolation was associated with an increased length of stay (11.8 vs 7.5 days). In a prospective study from the UK, 37% of patients isolated *A. fumigatus* at baseline at steady state. Isolation of *A. fumigatus* was associated with higher inhaled corticosteroid dose and higher sputum total cell and neutrophil count. However, there was no link with exacerbation frequency or FEV1 [119]. The association with a higher dose of inhaled corticosteroids was also shown in a retrospective pair-matched study from China; patients with *Aspergillus* isolation had more prolonged hospital stay (15 vs 12 days) [141]. Finally, patients with COPD who reported activities related to fungal exposure reported more exacerbations. This may suggest a potential association with *Aspergillus* exposure [142].

## Pathogenesis of aspergillosis in COPD

The pathogenesis of COPD involves infections, chronic inflammation and oxidative stress [143]. *A. fumigatus* is the most common pathogen isolated from the sputum of patients with COPD at steady state and during exacerbations [14]. The phagocytosis of bacteria and apoptotic cells by the alveolar macrophages in COPD patients is impaired though it is uncertain if this applies to the phagocytosis of *Aspergillus* conidia [144]. Patients with COPD are thought to have impaired ciliary function resulting from exposure to cigarette smoke and recurrent chest infections. This may promote the binding of the *Aspergillus* conidia to the airways in a concentration-dependent manner. The epithelial responses to *A. fumigatus* result in remodelling of the respiratory epithelium with a heightened expression of Th2 cytokines [13]. Cytoskeletal collapse and apoptosis eventually occur. In COPD, it is possible that a combination of several factors limits the ability to eliminate the conidia of *Aspergillus*. Defects of the innate immune system (inherited or acquired), regular use of high-dose steroids in addition to frequent use of broad-spectrum antibiotics in case of exacerbations, may all play a role in promoting the colonisation of the respiratory epithelium by *Aspergillus.*

Pentraxin 3 (PTX3) is an acute phase protein released during inflammation or injury by several cells. PTX3 binds to the conidia of *A. fumigatus* and serves as an opsonising agent [145] while also triggering the classical complement pathway and mediating innate immunity to *A. fumigatus* [146–148]. A recent study carried out among a Chinese COPD population showed a significant association between PTX3 rs1840680 single nucleotide polymorphisms and the susceptibility to pulmonary aspergillosis [149]. Plasma PTX3 levels have been found to substantially increase in the setting of fungal infection [150] and may have utility in the diagnosis of IA in COPD patients.

There is growing evidence that severe COPD predisposes to the development of IA especially, but not exclusively, in the setting of steroid use [31, 32, 37, 39, 48]. It is still unclear why some COPD patients get colonised by *Aspergillus* spp., whereas others develop IA. High doses of oral steroids reportedly promote the growth of *Aspergillus* [151] while enhancing Th2 cytokines and dampening Th1 cytokine response [152]. Steroids also inhibit neutrophil action and decrease alveolar macrophage antifungal activity by limiting the production of reactive oxidant intermediates [153, 154]. In vitro studies suggest that steroids promote the growth of *A. fumigatus* as 30–40% increase in growth rate of *A. fumigatus* and *A. flavus* exposed to pharmacological doses of hydrocortisone was demonstrated [151]. The exact mechanism for this increased growth is not fully understood, but the presence of a ligand/receptor system in the fungi has been proposed as a putative mechanism for this association.

A retrospective study of 239 patients admitted with COPD with *Aspergillus* isolation identified the accumulated dosage of corticosteroids equivalent to > 700 mg prednisolone in the three months prior to admission to be an independent predictor of IA [37]. In this same study, the receipt of antibiotic treatment in the 3 months prior to admission was also a predictor of IA. Inhaled corticosteroids have also been identified as a risk factor for IA [155]. A study conducted among 11 patients with COPD from which *Aspergillus* isolates were cultured from lower respiratory tract samples revealed that 8 (72.7%) of the patients had used an inhaled steroid, while 10 (90.9%) had used a systemic steroid within the last 3 weeks [41]. 

A retrospective case–control study of 30 COPD patients with IA and 60 COPD control patients without IA in China identified treatment with three or more antibiotics during hospitalisation and antibiotic treatment longer than 10 days to be risk factors for IA [40]. The use of broad-spectrum antibiotics in the setting of COPD is thought to disrupt the normal flora of the respiratory epithelium thereby promoting the proliferation of fungal pathogens such as *Aspergillus* [156]. The ability of *Aspergillus* to form biofilms on bronchial epithelial cells which are difficult to eradicate with antifungals may also be a contributory factor [157]. 

## Uncertainties, future research

Further research is required in several areas. Optimum means of diagnosing IA in COPD with sputum culture/PCR, *Aspergillus* antibody and antigen, BDG and identification of new biomarkers are key areas to explore further. Prospective studies are needed to establish the significance of *Aspergillus* isolation in COPD exacerbations, including intervention studies, and consensus reached on whether this requires treatment. The utility of *Aspergillus* biomarkers such as BDG and *Aspergillus* antibody assays in COPD monitoring of therapy for IA in COPD needs to be clearly elucidated.

Likewise, genetic risk factors for IA in need to be investigated. There are many described in leukaemia, haematopoietic stem cell transplantation and solid organ transplantation, but this is largely unexplored in COPD. In addition to PTX3 polymorphisms, other genetic defects may be implicated, such as those affecting the Th1 cytokine pathway. The role of fungal exposure in the development of CPA in patients with COPD also needs to be better defined. The efficacy of IFNγ immunotherapy in the setting of CPA/COPD overlap remains an underexplored but promising area of research.

In conclusion, COPD poses a significant global health concern and urgent measures are required to mitigate the current situation. Healthcare professionals need to be aware of the risk of IA and various forms of aspergillosis in their COPD patients to aid early diagnosis, facilitate prompt management and reduce mortality.

## Data Availability

Not applicable.
